# Clinical Features and Natural History in a Cohort of Chinese Patients with RPE65-Associated Inherited Retinal Dystrophy

**DOI:** 10.3390/jcm10225229

**Published:** 2021-11-10

**Authors:** Jie Shi, Ke Xu, Jian-Ping Hu, Yue Xie, Xin Zhang, Xiao-Hui Zhang, Zi-Bing Jin, Yang Li

**Affiliations:** Beijing Ophthalmology & Visual Sciences Key Lab, Beijing Institute of Ophthalmology, Beijing Tongren Eye Center, Beijing Tongren Hospital, Capital Medical University, Beijing 100730, China; sjyy9913@163.com (J.S.); xuke1870@163.com (K.X.); pucri_hujp@126.com (J.-P.H.); moonxieyue@163.com (Y.X.); 15564045436@163.com (X.Z.); xhzhang711@gmail.com (X.-H.Z.)

**Keywords:** *RPE65*-associated inherited retinal dystrophy, clinical features, *RPE65* variants, genotype, best-corrected visual acuity

## Abstract

*RPE65*-associated inherited retinal dystrophy (*RPE65*-IRD) is an early-onset retinal degeneration. The aim of this study was to describe the clinical features and natural course of this disease in a Chinese patient cohort with *RPE65* biallelic variants. Thirty patients from 29 unrelated families with biallelic disease-causing *RPE65* variants underwent full ophthalmic examinations. Thirteen were followed up over time. An additional 57 Chinese cases from 49 families were retrieved from the literature to analyze the relationship between best-corrected visual acuity (BCVA) and age. Our 30 patients presented age-dependent phenotypic characteristics. Multiple white dots were a clinical feature of young patients, while maculopathy, epiretinal membrane, and bone spicules were common in adult patients. Among the 84 patients, BCVA declined with age in a nonlinear, positive-acceleration relationship (*p* < 0.001). All patients older than 40 years met the WHO standard for low vision. Longitudinal observation revealed a slower visual acuity loss in patients younger than 20 years than those in their third or fourth decade of life. Our study detailed the clinical features and natural course of disease in Chinese patients with *RPE65*-IRD. Our results indicated that these patients have a relatively stable BCVA in childhood and adolescence, but eyesight deteriorates rapidly in the third decade of life. These findings may facilitate the implementation of gene therapy in China.

## 1. Introduction

The retinal pigment epithelium-specific 65 kDa protein (RPE65) is a retinoid isomerohydrolase encoded by the *RPE65* gene (OMIM 180069). This gene is preferentially and abundantly expressed in the retinal pigment epithelium (RPE) [1], where it is responsible for an essential enzymatic step that converts all-trans retinal ester to 11-cis retinol in the visual cycle—a conversion that is critical for visual pigment formation in photoreceptors [1]. The *RPE65* gene contains 14 coding exons spanning 20 kb and is localized on chromosome 1p31. Biallelic causal variants in RPE65 can cause certain inherited retinal dystrophies (IRD), such as Leber’s congenital amaurosis (LCA) and early-onset severe retinal dystrophy (EOSRD) [1,2,3]. LCA has a severer phenotype and an earlier onset age than EOSRD. The former usually presents within a few months after birth, while the latter develops at between 1 and 5 years of life [4]. The mutation frequency of RPE65 in LCA patients varies from 1.7% to 16% depending on patient race or geographical origin [2,3,5]. Several previous studies have indicated that variants of *RPE65* were responsible for about 3.0–7.7% of Chinese patients with LCA and 10.5% of the patients with EOSRD [6,7,8]. To date, 257 disease-causing variants in *RPE65* have been reported (HGMD Professional 2020.4), but no explicit genotype and phenotype relationships have been reported in several previous published studies, and only a few studies have reported that patients with hypomorphic missense variants showed milder phenotypes [6,9,10,11,12,13].

Recently, phase III clinical trials of gene augmentation therapy have shown promising and successful results in *RPE65*-associated inherited retinal dystrophy (*RPE65*-IRD) cases, and Luxturna (voretigene neparvovec-rzyl) was approved in 2017 by the U.S. Food and Drug Administration as the first gene therapy for IRD patients [14,15,16]. The oral visual-cycle modulators, including 9-cis-retinyl-acetate (QLT091001, zuretinol), oral retinoid, and 9-cis-ß-carotene, are also under investigation and may slow down vision loss by restoring 11-cis-retinal levels in *RPE65*-IRD [17]. These discoveries highlight the importance of investigating the natural course of *RPE65*-IRD diseases for the determination of a time window for gene therapy or for evaluating therapeutic effects.

Previous studies in Chinese patients have mainly focused on descriptions of the frequency or genetic findings of *RPE65* in patients with IRD, but they lack any detailed description of the phenotypic spectrum and natural course of this progressive disease [6,7,8,18]. In the current study, we delineated the clinical features and natural course of visual acuity changes in 30 patients from 29 unrelated Chinese families carrying molecular-confirmed biallelic variants of *RPE65* by cross-sectional and longitudinal investigation. We then combined the data from those 30 patients with data from 57 Chinese patients from eight previously published studies to further determine the relationship between BCVA and age in Chinese patients with *RPE65*- IRD. These results could provide guidance for future gene therapy in Chinese patients.

## 2. Subjects and Methods

### 2.1. Subjects and Clinical Evaluation

This retrospective study was performed according to the tenets of the Declaration of Helsinki for research relating to human subjects and was approved by the Beijing Tongren Hospital Joint Committee on Clinical Investigation. Informed written consent was obtained from all patients before their enrollment in this study. A total of 30 patients from 29 unrelated families harboring biallelic RPE65 variants were recruited from the Genetics Laboratory of the Beijing Institute of Ophthalmology from February 2012 to October 2020. Of these, 16 patients had been described in our previous studies [7,19], and 13 patients were followed up.

Each participant underwent routine ophthalmic examinations, including best-corrected visual acuity (BCVA) using Snellen charts, slit-lamp biomicroscopy, and fundus photography; the exceptions were three patients (under 6 years old) who did not have a BCVA test. Most patients underwent optical coherence tomography (OCT, Spectralis, Heidelberg Engineering, Heidelberg, Germany), fundus autofluorescence (FAF, Heidelberg Engineering, Heidelberg, Germany), and full-field electroretinography (ERG, Roland, Germany). ERG recording followed the standard protocol of the International Society for Clinical Electrophysiology of Vision (ISCEV). All of the fundus imaging and ERG were evaluated by two doctors (one junior and one senior qualified retinal-ophthalmologists). We stratified the patients into four groups based on their last presenting age as follows: patients in groups 1, 2, 3, and 4 were individuals aged 0–10, 11–20, 21–30, and >30 years, respectively.

### 2.2. Genetic Testing and Bioinformatics Analysis

We performed targeted exome sequencing (TES) in the 29 probands using a capture panel consisting of 188 known inherited retinal degeneration genes, previously developed and evaluated by our group [20]. The Illumina library preparation and capture experiment were performed as previously described [20]. Two databases, the HGMD database (http://www.hgmd.cf.ac.uk/ac/index.php) and the LOVD database (https://grenada.lumc.nl/LOVD2/eye/home.php), were used to search for reported pathogenic variants. The pathogenicity of the variants was predicted by three in silico programs: PolyPhen2 (http://genetics.bwh.harvard.edu/pph, in the public domain), Mutation Taster (http://www.mutationtaster.org, in the public domain), and SIFT (http://sift.jcvi.org/, in the public domain). The programs NetGene2 Server (http://www.cbs.dtu.dk/services/NetGene2/), Alternative Splice Site Predictor (ASSP, http://wangcomputing.com/assp/index.html), and Berkeley Drosophila Genome Project (BDGP, http://www.fruitfly.org/seq_tools/splice.html) were used to analyze any variants involving a splicing effect. Co-segregation analysis was performed whenever the DNA of any family members was available.

### 2.3. Statistical Analysis

We combined 27 patients from our own cohort with 57 Chinese patients from eight previously published papers to determine the relationship between BCVA and age in Chinese patients with RPE65-IRD [6,8,18,21,22,23,24,25]. The additional Chinese patients with biallelic *RPE65* variants were selected by searching key words “RPE65 and Chinese or China” in the PubMed directory. For some patients who were repeatedly described in different studies, we chose the updated version. The patients with only one *RPE65* variant detected or those without available BCVA were excluded. The eight studies described the age, BCVA, and variants in the RPE65 for 57 patients from 49 unrelated families (Table S1). We converted the Snellen ratios into the logarithm of the minimum angle of resolution (logMAR) values for statistical purposes. The logMAR values of 0, 1.0, 1.85, 2.3, and 2.7 are equal to a Snellen decimal vision of 1.0, 0.1, counting fingers, hand movements, and light perception, respectively. Mixed-effects linear/polynomial regression models implemented via maximum likelihood were applied to determine any potential association between age and BCVA. A one-way ANOVA, followed by Tukey HSD, was adopted to compare the differences in BCVA between the groups. We chose the BCVA of the better-seeing eye for each patient to perform the statistical analysis except longitudinal analysis, in which the data of both eyes were assessed due to the change variation between both eyes. A value of *p* < 0.05 was considered statistically significant.

## 3. Results

### 3.1. RPE65 Variants

We detected 41 distinct disease-causing variants of RPE65 in the 29 unrelated families. The variants included 25 missense, seven frameshift indel, six nonsense, and two splicing effect variants, as well as one in-frame deletion variant. Of these 41 variants, 10 were first found in the current study. These 10 novel variants comprised four missense, three frameshift small indel, two splice effect, and one nonsense variants. None of these novel variants was previously recorded in any public database, and all were defined as pathogenic or likely pathogenic based on the American College of Medical Genetics and Genomics (ACMG) guidelines and standards (Table S2).

**Figure 1:**
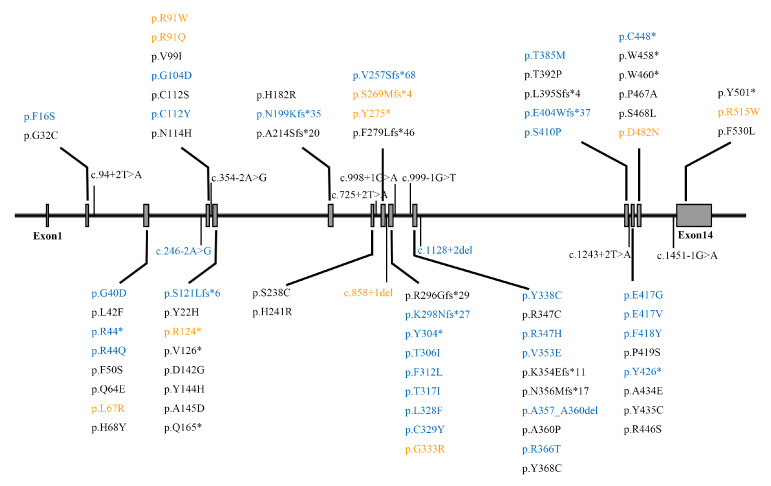
The distribution of 85 distinct variants of RPE65 identified in a Chinese cohort including 78 unrelated families. Blue: variants only identified in 29 families of our own cohort. Black: variants reported in pedigrees from eight previously published papers. Yellow: variants identified or reported in both our own cohort and the eight previously published papers; * a stop codon (http://varnomen.hgvs.org/recommendations/protein/variant/substitution/).

In total, 85 disease-causing variants of RPE65 were detected or reported in the large cohort including our own 29 pedigrees and 49 pedigrees from the eight previously published papers. These variants included 51 missense, 13 frameshift indel, 11 nonsense, and nine splicing effect variants, as well as one in-frame deletion variant. Their distribution in the RPE65 gene is displayed in Figure 1.

### 3.2. Patient Demographics

Of the 30 patients from the 29 families, 18 individuals were diagnosed with LCA and 12 had EOSRD. The mean onset age range was 1.57 ± 1.59 years (range: 0.1–5.0 years; median, 0.75 years). The mean age at the last presentation was 18.06 ± 13.37 years (range: 1.0–45.0 years; median, 15.25 years). Eighteen patients were males. Most patients experienced night blindness, photoattraction, or different extents of visual defects. About 65% of the patients had nystagmus. Their detailed clinical features are summarized in Table 1.

### 3.3. The Relationship between Visual Acuity and Age

**Figure 2:**
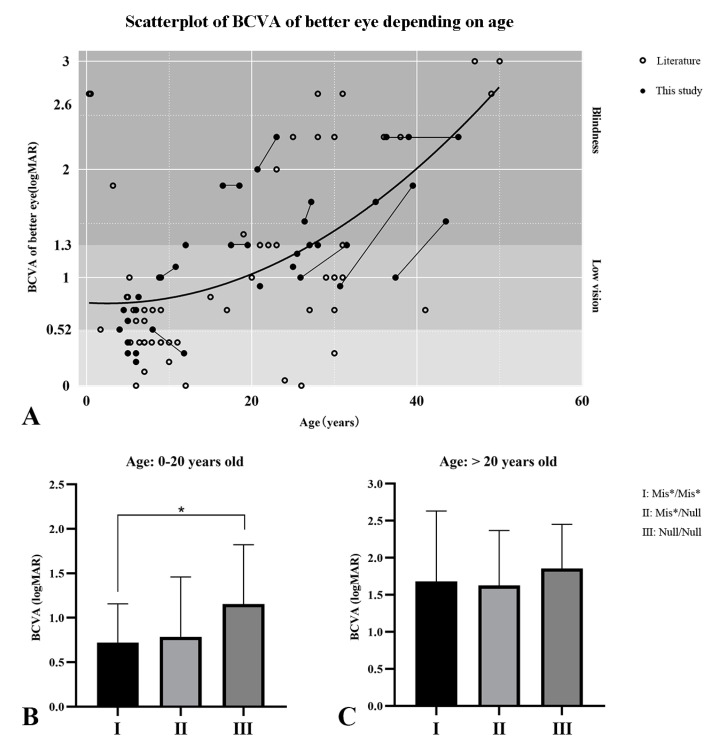
The best-corrected visual acuity (BCVA) for the better-seeing eye as a function of age, and the relationship between BCVA and genotype. (**A**) The BCVA as a function of age in the 84 better-seeing eyes of 84 patients with RPE65-IRD, including the 27 patients in our cohort (black dots) and 57 patients from eight previously published Chinese studies (black circles). When serial data were available (*n* = 10), visual acuities from the same eye at the first and the latest evaluation were plotted and connected by lines. The thicker line is the quadratic function of the best fit. (**B**,**C**) Comparison of the BCVA of patients with different genotypes in two age groups. For patients under 20 years of age (**B**), the numbers of patients with Ⅰ, Ⅱ and Ⅲ genotypes were 26, 12, and 9, respectively; for patients older than 20 years (**C**), these numbers were 16, 16, and 5, respectively. A significant difference was found between Ⅰ and Ⅲ genotypes in the patients under 20 years of age (* *p* < 0.05). No difference was found among other subgroups. Mis*: missense or in-frame deletion variant. Null: null variant.

The mean BCVA (log MAR) of the 27 patients at the last time of examination was 1.16 ± 0.63 (range 0.22–2.30). The mean BCVA values for groups 1, 2, 3, and 4 were 0.51 ± 0.20, 1.17 ± 0.50, 1.41 ± 0.43, and 1.83 ± 0.37, respectively (*p* < 0.001; *p* (2 vs. 3) = 0.751; *p* (2 vs. 4) = 0.058; *p* (3 vs. 4) = 0.261). We combined the 27 patients in our current cohort with the 57 Chinese patients from eight previously published studies to obtain a more precise relationship between BCVA and age in Chinese patients with RPE65-IRD. The mean age of the 84 patients was 17.96 ± 13.11 years (range 0.3–50), and their average BCVA (LogMAR) was 1.20 ± 0.81 (range 0–3.00). The process for determining BCVA for the better-seeing eye as a function of age is presented in Figure 2A. The BCVA decreased with age in a nonlinear, positive-acceleration relationship (*p* < 0.001) (Figure 2A). Nevertheless, visual acuity remained relatively stable in childhood and adolescence but descended rapidly in the third decade of life. Half of these subjects were blind according to WHO criteria at the third decade of life, and all the patients met the standard of low vision when they were more than 40 years old.

We also investigated the relationship between BCVA and genotype in the large cohort of 84 patients. For the patients under 20 years, the mean BCVA was significantly worse for the patients carrying two null allele variants (1.15 ± 0.65 log MAR) than for the patients harboring two missense variants (0.72 ± 0.43 log MAR) (Figure 2B). For the patients over 20 years of age, no significant difference was found in mean BCVA among the patients with different genotypes (Figure 2C).

### 3.4. Fundus Features

**Figure 3:**
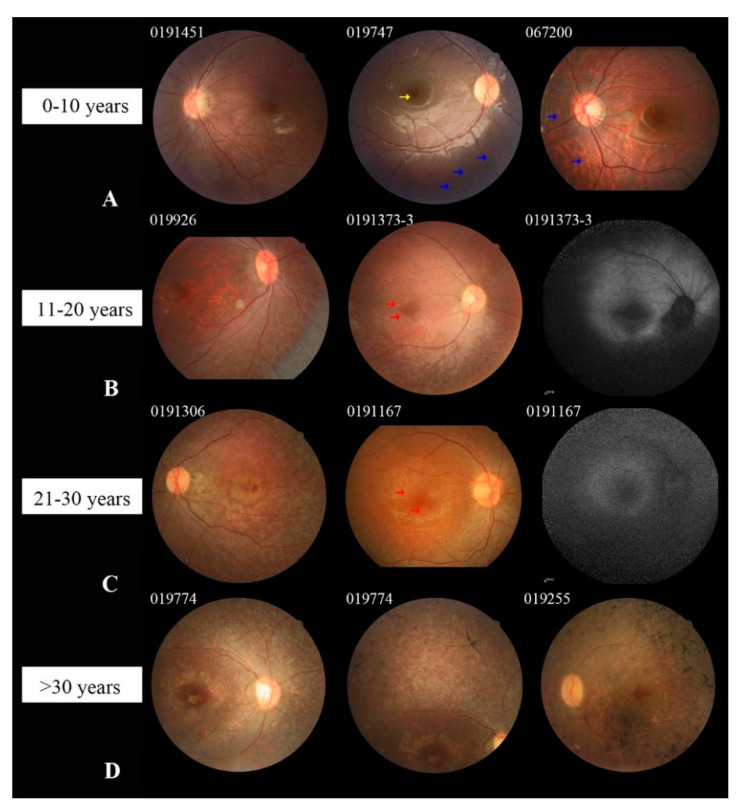
Colored fundus (CF) and fundus autofluorescence (FAF) photographs of patients with RPE65-IRD. (**A**) CF photographs of three patients in group 1 show tapetoretinal degeneration (TD), white dots (WD, marked by blue arrows) in the mid-peripheral retina, and yellowish granular dots (yellow arrows) in the fovea. CF image of patient 067200 displays diffuse WD with a leopard fundus. (**B**) CF photographs of patients in group 2 show obvious TD and macular atrophy, and the epiretinal membrane in the macula (red arrow). FAF of patient 0191373-3 displays extensive hypo-autofluorescence and a parafoveal hyper-autofluorescence ring. (**C**) CF and FAF images of patients in group 3 showed extensive chorioretinal atrophy. (**D**) CF photographs of patients in group 4 presented with severe and extensive TD, macular atrophy, and bone-spicule-like pigment deposits.

**Figure 4:**
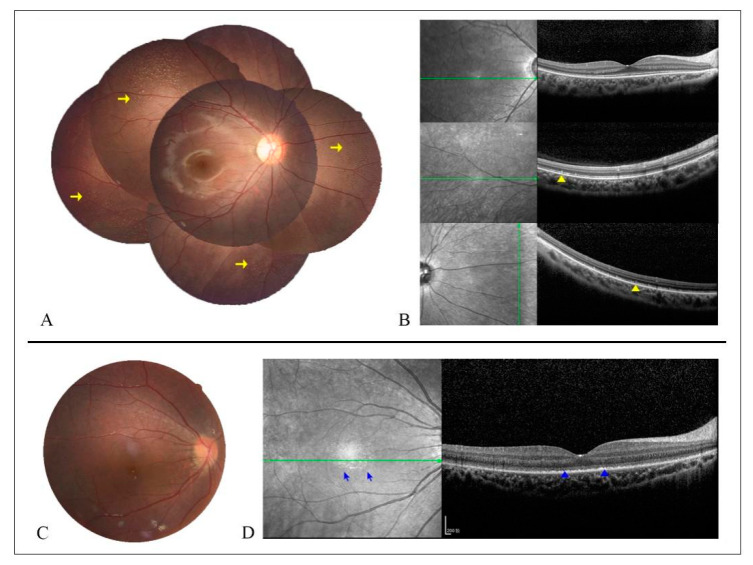
Colored fundus (CF) images and OCT scans in patients 0191010 (**A**,**B**) and 0191577 (**C**,**D**). (**A**) CF image shows abundant white dots (WDs) in the mid-peripheral and peripheral retina (yellow arrow). (**B**) OCT scans display hyperreflective remnants (yellow triangle) corresponding to the WDs. (**C**) CF photography shows an almost normal fundus appearance. (**D**) Infrared fundus image reveals multiple drusen-like fine granular dots in the fovea (blue arrow), which presented as a diffuse hyperreflective substance in the retinal pigment epithelium (RPE) layer in the OCT image (blue triangle).

Among our 30 patients, 29 subjects (57 eyes) had clear fundus photographs. Twelve patients in group 1 showed mild tapetoretinal degeneration (TD), mainly in the peripheral and mid-peripheral retina, and five of them also presented with multiple white dots (Figure 3A). The multiple white dots were mainly distributed in the peripheral or mid-peripheral retina, with some extending to the vascular arcades or involving the posterior pole (Figure 3A and Figure 4A). Fine yellowish granular dots in the fovea were detected in three patients; these were more evident in the infrared fundus images than in the color photographs (Figure 3A and Figure 4C,D). No fundus autofluorescence (FAF) images were available in this group of patients because of their young age and nystagmus. Five patients in group 2 showed obvious TD and four of them presented macular atrophy and epiretinal membrane; the exception was patient 0191010. FAF images in these five patients demonstrated extensive hypo-AF in the peripheral and mid-peripheral retina, with an abnormal hyper-AF ring in the parafoveal area (Figure 3B). Six patients (11 eyes) in group 3 and six patients in group 4 showed severe and extensive TD and macular atrophy (Figure 3C,D). FAF images in these 12 patients showed a similar fluorescence pattern to that observed in group 2, but with a relatively smaller hyper-AF ring or a half hyper-AF ring. Bone spicules were found in all patients in groups 3 and 4, and these became denser and clumped with aging (Figure 3C,D).

### 3.5. Central Retinal Structural Features Evaluated by SD-OCT

**Figure 5:**
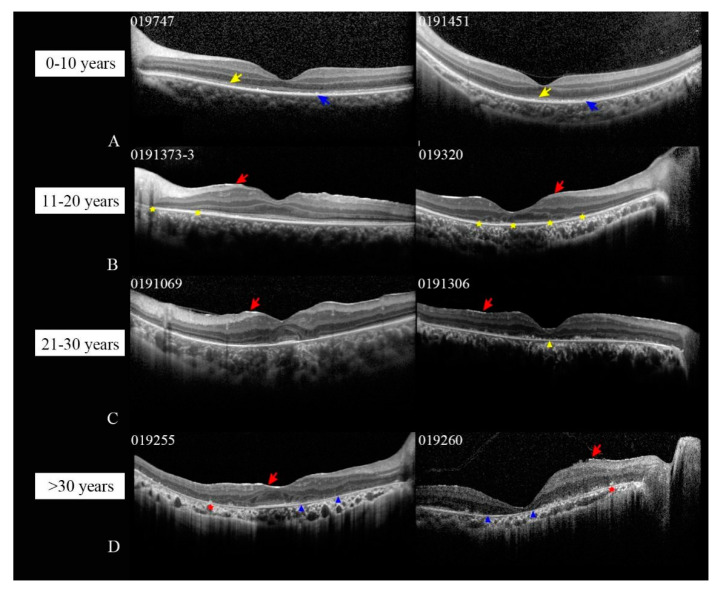
Spectral domain OCT scans in patients with RPE65-IRDs. (**A**) OCT scans of patients 019747 and 0191451 in group 1 (age 0–10 years) show a coarse ellipsoid zone (EZ; yellow arrow) and hyperreflective remnants in the retinal pigment epithelium (RPE; blue arrows). (**B**) OCT scans of patients 0191373-3 and 019320 in group 2 (age 11–20 years) showed a distorted inner retinal architecture with a disruption and absence of the EZ in the foveal and parafoveal regions, respectively (yellow asterisk). (**C**) OCT scans of the patients in group 3 (age 21–30 years) exhibited distorted retinal architectures (0191069), atrophy in the fovea (yellow triangle), and disruption of the EZ. (**D**) OCT images of patients in group 4 (age > 30 years) revealed a disorganized macular structure with destruction of the IS/OS and RPE (blue triangle) and hyperreflective depositions in the outer retina (red asterisk). Epiretinal member (red arrow) presented in all patients from groups 2–4.

We performed SD-OCT scanning in 20 patients (39 eyes) in our own cohort, including five patients in group 1, five patients in group 2 (including patient 019320 whose age is 10.8 years old), four patients in group 3, and six patients in group 4. For the five patients in group 1, OCT scans showed a well-preserved retinal lamellar architecture with a coarse ellipsoid zone (EZ) and hyperreflective remnants in the RPE (Figure 4B and Figure 5A). OCT examinations of the patients in group 2 displayed distorted inner retinal architectures, with a disruption and absence of the EZ in the foveal and parafoveal regions, respectively. Foveal hypoplasia was observed in three patients (Figure 5B). OCT scans of the patients in group 3 exhibited foveal hypoplasia, distorted retinal architectures or atrophy in the fovea, and absence of the EZ in the parafoveal regions (Figure 5C). The OCT scans of the patients in group 4 revealed extensive disorganized macular structures or atrophy with destruction of the photoreceptor inner/outer junctions (IS/OS) and RPE and hyperreflective depositions in the outer retina (Figure 5D). Foveal hypoplasia was noted in one patient (019255). The epiretinal membrane was observed in all eyes of the patients in groups 2–4 (Figure 5B–D), except for patient 0191010.

### 3.6. Longitudinal Observation of the 13 Followed Patients

**Figure 6:**
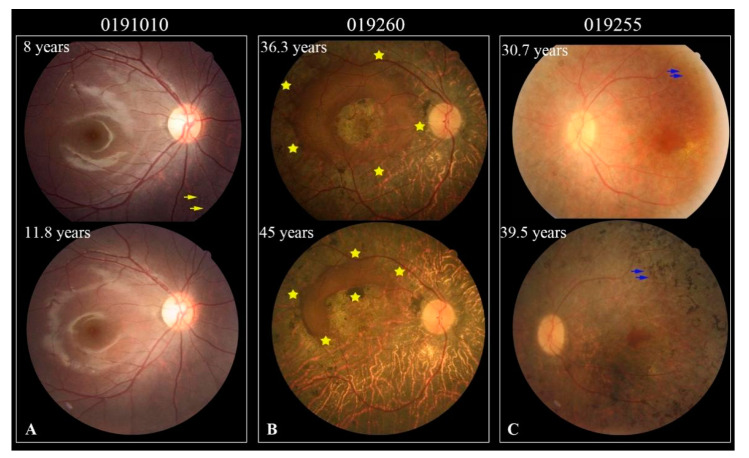
Colored fundus (CF) photographs of three patients during their follow-up. (**A**) CF photographs of patient 0191010 show that some white dots (yellow arrow) disappeared about three years later. (**B**) CF images of patient 019260 display an atrophy enlargement in the posterior pole during nearly nine years of follow-up. The relatively normal retina is surrounded by yellow asterisks. (**C**) CF photographs of patient 019255 show an increase in bone spicules (blue arrow) with aging.

We followed 13 patients longitudinally using fundus photography, central retinal OCT scanning, and FAF examination to monitor their BCVA and fundus appearance (Table 2). Three patients did not have BCVA values for their first visit due to their young age. The mean follow-up was 52.1 months (range, 6–104 months). The longitudinal analysis demonstrated a slow loss of VA, with a mean change rate of 0.067 logMAR/year. We noticed that the BCVA of the patients younger than 20 years was either stable or slightly improved, with a mean change rate of −0.052 logMAR/year. By contrast, the BCVA of the patients older than 20 years presented a discernible decline in the mean change rate of 0.126 logMAR/year. Fundus examination revealed that some white dots in the retina decreased or disappeared, while retinal and RPE atrophy in the posterior pole and bone spicules enlarged or increased with aging during the follow-up (Figure 6).

## 4. Discussion

In this study, we characterized the natural course of *RPE65*-IRD in a cohort of Chinese patients by both cross-sectional and longitudinal observations. We established a relatively accurate association between BCVA and age in 84 Chinese patients carrying biallelic RPE65 variants (27 patients in our own cohort and 57 patients from eight previously published studies). These findings might facilitate the implementation of gene therapy in China.

Visual acuity is considered an important factor for following patients with *RPE65*-IRD; however, no studies on BCVA variations with age have been performed previously in Chinese patients. Our cross-sectional and longitudinal data indicated that the BCVA of these patients was moderately stable during the first two decades of life but then decreased rapidly in the third decade of life. These findings were consistent with results observed in Caucasian patients [9,10,11,26] and further implied that the first two decades of life were a better intervention window for gene therapy. Two previous studies reported that patients carrying hypomorphic missense variants presented relatively mild phenotypes and good visual function [12,13]. Our results revealed that visual impairment was milder in patients with biallelic missense variants than in patients with biallelic null variants in the early stage. Previous in vitro functional analyses also indicated that some missense variants still retained 2.5–13.5% of their original RPE65 isomerase activity [13]. These residual levels of RPE65 activity may be sufficient to maintain partial functioning of photoreceptors in the early stage, but as the disease progresses, the residual isomerase activity no longer suffices to prevent slow cumulative degeneration. Our longitudinal observations indicated a transient, slight improvement of VA in two patients (019320 and 0191010), aged 11 and 12 years, respectively. This type of improvement in VA has also been reported in other LCA patients, and it might reflect a learning effect due to childhood maturation and development or test–retest variation [3].

Fundus examination of the patients in our own cohort presented age-dependent phenotypic characteristics. We detected white dots in six patients all under 12 years of age, and we observed that some of these white dots decreased or disappeared with aging during the follow-up. The white dots were easy to overlook when they were located in the peripheral retina; therefore, their prevalence in our cohort might be underestimated. The presence of white dots in patients with RPE65-IRD has been reported many times previously [6,8,12,27,28,29], and most patients with white dots were teenagers; therefore, we conjectured that the white dots might be a typical fundus manifestation of RPE65-IRD in the early stage but that they disappear over time. We did not perceive any association between the presence of white dots and either genotype or the extent of visual impairment. Consistent with two previous Chinese studies [6,8], we found maculopathy and bone spicules as common features in adult patients; however, bone spicules were not common in Caucasian patients at any age [10].

Our SD-OCT scans revealed that patients in their first decade of life had a relatively normal macular lamellar structure and disruption or defects in the EZ and RPE in the parafoveal regions. Kumaran et al. found a statistically significant correlation between the EZ width and BCVA [30]. In that study, which included 26 patients (age range 5–24 years), they noted foveal hypoplasia in half their cohort and found that patients with foveal hypoplasia had poor BCVA [30]. We also found foveal hypoplasia in eight patients in our own cohort, but all these patients had epiretinal membrane in the macula. We conjectured that the foveal hypoplasia might be related to the traction of the epiretinal membrane in the macula; however, this requires further observation in the future. The epiretinal membrane was observed in almost all patients over 11 years of age in our own cohort. This membrane is a common feature in patients with IRD and indicates advanced-stage rather than early-stage disease [31]. Therefore, changes in the central retinal structures might be used as other parameters for monitoring disease progression in patients with RPE65-IRD.

The current study has several limitations. One is its retrospective design and another is that the number of patients in our own cohort is relatively small. We also did not conduct any quantitative analysis for OCT, so we cannot accurately describe the characteristics of the changes in the patient images.

In conclusion, our study described in detail the clinical features and the natural course of disease for Chinese patients with *RPE65*-IRD. Our results indicated that the patients have a relatively stable visual acuity throughout childhood and adolescence, but the BCVA declines rapidly in the third decade of life. These findings may facilitate the implementation of gene therapy in China.

## Figures and Tables

**Table 1:** The clinical features and results of the RPE65 gene variant screening of the patients.

Patient ID	Diagnosis	Gender	Age (Year)		Visual Acuity (logMAR) OD/OS		Initial Symptom	Nystag-mus	Fundus Apperence OU or; OD/OS	ERG	Variants		Co-Segregation
			Onset	Exam							Allele1	Allele2	
0191288	LCA	M	0.4	1.0	NA	NA	NB	Yes	TD	SD	p.(Cys448*)	p.(Ser269Metfs*4)	Yes
019390 †	EOSRD	M	2.0	4.0	0.52	0.52	NB	No	TD, WD	NA	p.(Asp482Asn)	p.(Arg515Trp)	Yes
0191577	LCA	F	0.1	4.5	0.70	0.70	NB	Yes	TD	EX	p.(Thr317Ile)	p.(Gly333Arg)	Yes
019227 †	EOSRD	M	3.0	5.0	NA	NA	NB	No	TD	NA	p.(Lys298Asnfs*27)	p.(Leu67Arg)	Yes
019690	EOSRD	F	4.0	5.0	0.30	0.70	NB	NA	TD, WD	NA	p.(Phe312Leu)	p.(Phe312Leu)	Yes
0191451	LCA	M	0.5	5.0	0.60	0.70	PA	Yes	TD	SD	p.(Glu404Trpfs*37)	c.1128 + 2del	Yes
067200 †	LCA	M	0.5	5.0	0.52	0.40	NB	No	TD, WD #	NA	p.(Leu328Phe)	p.(Tyr275*)	Yes
0191647	LCA	F	0.1	5.3	NA	NA	PA	Yes	TD	EX	p.(Leu67Arg)	p.(Asn199Lysfs*35)	Yes
019602	EOSRD	M	3.0	6.0	1.00	0.70	NB	NA	TD	EX	p.(Arg91Trp)	p.(Arg91Trp)	Yes
A302	EOSRD	M	1.0	6.0	0.30	0.30	NB	NA	TD	NA	p.(Thr306Ile)	p.(Tyr304*)	Yes
019247 †	LCA	M	1.0	6.0	0.22	0.40	NB	No	TD, WD	SD	p.(Thr385Met)	p.(Thr306Ile)	Yes
019747 †	LCA	M	0.5	6.3	1.00	0.82	NB	No	TD, WD	EX	p.(Phe16Ser)	p.(Ser121Leufs*6)	Yes
019320 †	LCA	F	0.3	10.8	1.10	1.10	PA	Yes	TD, MD, EM	EX	p.(Arg44*)	p.(Glu417Gly)	Yes
0191010 †	EOSRD	F	3.0	11.8	0.30	0.40	LV	No	TD, WD	SD	p.(Arg347His)	p.(Ala357_Ala360del)	Yes
019926 †	LCA	M	0.3	12.0	1.30	1.30	NB	Yes	TD;/EM	EX	p.(Glu417Val)	p.(Glu417Val)	Yes
0191373	LCA	M	0.6	18.5	1.85	1.85	LV	Yes	TD, EM	EX	p.(Leu67Arg)	p.(Leu67Arg)	Yes
0191373-3	LCA	M	0.9	19.5	1.30	1.40	LV	Yes	TD, EM	EX	p.(Leu67Arg)	p.(Leu67Arg)	Yes
A250	EOSRD	F	3.0	21.0	0.92	0.92	NB	NA	NA	NA	p.(Gly104Asp)	p.(Arg91Gln)	Yes
0191306	EOSRD	F	2.0	23.0	2.30	2.30	NB	Yes	TD, MD, EM	SD	p.(Tyr338Cys)	c.246-2A > G	Yes
019190 †	EOSRD	M	5.0	25.0	1.10	2.00	LV	Yes	TD, EM, BS	NA	p.(Val257Serfs*68)	p.(Leu67Arg)	Yes
0191167	EOSRD	F	5.0	25.5	1.22	1.22	NB	Yes	TD, EM, BS	EX	p.(Cys112Tyr)	p.(Cys112Tyr)	Yes
019481 †	LCA	F	0.5	27.0	1.30	1.30	NB	Yes	TD, EM, BS #	EX	p.(Arg44Gln)	c.858 + 1del	Yes
0191069 †	LCA	M	0.5	27.2	1.70	2.30	NB	Yes	TD, EM, BS;/MD	EX	p.(Val353Glu)	p.(Val353Glu)	Yes
0191536	LCA	M	0.5	28.0	2.70	1.30	LV	Yes	NA/TD, MD, EM, BS #	NA	p.(Arg91Trp)	p.(Arg91Trp)	Yes
019774 †	EOSRD	F	3.0	31.5	1.40	1.30	NB	No	TD, EM, BS;MD/	EX	p.(Ser410Pro)	p.(Arg91Gln)	Yes
0191211	LCA	F	0.1	35.0	1.70	2.00	NB	Yes	TD, EM, BS	EX	p.(Cys329Tyr)	p.(Cys329Tyr)	Yes
0191141	EOSRD	M	5.0	39.0	2.30	2.30	NB	Yes	TD, MD, EM, BS	EX	p.(Phe418Tyr)	p.(Gly333Arg)	Yes
019255 †	LCA	F	0.1	39.5	1.85	1.85	NB	No	TD, MD, EM, BS	NA	p.(Gly40Asp)	p.(Arg124*)	Yes
019245 †	LCA	M	1.0	43.5	1.85	1.52	LV	No	TD, MD, EM, BS	EX	p.(Tyr426*)	p.(Tyr426*)	Yes
019260	LCA	M	0.3	45.0	2.30	2.30	NB	Yes	TD, MD, EM, BS #	NA	p.(Arg124*)	p.(Arg366Thr)	Yes

Abbreviations: BCVA, best corrected visual acuity; BS, bone spicules; EM, epiretinal membrane; EOSRD, early onset severe retinal dystrophy; EX, extinguish (no detected waves are recorded); F, female; LCA, Leber congenital amaurosis; LV, low vision; M, male; MD, macular dystrophy; NA, not available; NB, night blindness; OD, right eye; OS, left eye; OU, bilateral eyes; PA, photoattraction; SD, severe damage (the amplitude is lower than the normal value by at least 70%); TD, tapetoretinal degeneration; WD, white dot deposits; * a stop codon (http://varnomen.hgvs.org/recommendations/protein/variant/substitution/); # leopard fundus, † in our previously published study footer.

**Table 2:** The follow-up information and clinical features of patients with biallelic RPE65 variants in this study.

Patient ID	Gender	Age (Year)		Follow-Up Interval (Month/Year)	VA (logMAR, OD/OS)		Fundus Feature	
		First	Last		Fisrt	Last	Fisrt	Last (Change)
0191577	F	4.0	4.5	6/0.5	NA/NA	0.70/0.70	TD	Unremarkable
0191451	M	3.2	5.0	21/1.8	NA/NA	0.60/0.70	TD	Unremarkable
019747	M	0.8	6.3	66/5.5	NA/NA	1.00/0.82	TD, WD	WD decreased
019320	F	2.5	10.8	100/8.3	1.40/1.40	1.10/1.10	NA	TD, MD, EM
0191010	F	7.8	11.8	48/4.0	0.52/0.52	0.30/0.40	TD, WD	WD decreased
0191373	M	16.5	18.5	24/2.0	1.85/1.85	1.85/1.85	TD, EM	Unremarkable
0191373-3	M	17.5	19.5	24/2.0	1.30/1.30	1.30/1.40	TD, EM	vascular sheath
0191306	F	20.7	23.0	28/2.3	2.00/2.00	2.30/2.30	TD, MD, EM	Sporidic BS
0191069	M	26.3	27.2	11/0.9	1.52/2.00	1.70/2.30	TD, EM, BS;/MD	Unremarkable
019774	F	25.9	31.5	67/5.6	1.00/1.00	1.40/1.30	TD, MD, EM	Sporidic BS
019255	F	30.8	39.5	104/8.7	0.92/1.00	1.85/1.85	TD, MD, EM, BS	BS and MD increased
019245	M	37.3	43.5	74/6.2	1.85/1.00	1.85/1.52	TD, MD, EM, BS	BS and MD increased
019260	M	36.3	45.0	104/8.7	2.30/2.30	2.30/2.30	TD, MD, EM, BS	BS and MD increased

Abbreviations: BCVA, best corrected visual acuity; BS, bone spicule; F, female; M, male; MD, macular atrophy; EM, epiretinal membrane; NA, not available; TD, tapetoretinal degeneration; WD, white dot deposits.

## Data Availability

Data is contained within the article or supplementary material.

## Supplementary Materials

The following are available online at https://www.mdpi.com/article/10.3390/jcm10225229/s1. Table S1: The clinical features and results of the RPE65 gene variants screening of the patients in the reported Chinese study, Table S2: Presumed pathogenic RPE65 variants identified in this study and analysis of the variants by predictive programs.
